# Pneumonic Injury and Repair: A Synopsis

**DOI:** 10.3390/ph16091255

**Published:** 2023-09-05

**Authors:** Nektarios Barabutis

**Affiliations:** School of Basic Pharmaceutical and Toxicological Sciences, College of Pharmacy, University of Louisiana Monroe, Monroe, LA 71201, USA; barabutis@ulm.edu; Tel.: +1-(318)-342-1460; Fax: +1-(318)-342-1737

It has been my great pleasure to have joined forces with *Pharmaceutical’s* editorial team in order to organize and publish a Special Issue on “Lung Injury and Repair”. The subject matter was carefully chosen so as to emphasize—and focus on—relevant works in pulmonary pathophysiology and lung disease treatment. The aforementioned topics are of the highest priority among the scientific and medical community since the corresponding complications (e.g., sepsis and lung injury) are associated with unacceptably high mortality rates [1,2]. Experts in the field were invited to contribute their works to this Special Issue, and fifteen articles of the highest quality were accepted for publication following rigorous evaluation. I would like to cordially express my gratitude to all involved parties, including both the authors and the reviewers. In the following section, I will summarize the important findings highlighted in this Special Issue.

Bronchoalveolar lavage fluid (BALF) proteome analysis identified critical targets involved in chronic obstructive pulmonary disease (COPD) pathogenesis, and drug candidates for COPD treatment were discovered [3]. A pilot study suggested that carbocysteine is involved in managing COPD—at least in part—due to its robust anti-inflammatory activities [4], while another paper reported that Wnt5a expression in airway smooth muscle cells led to aggravated fibrosis [5]. In the early stage of endotoxemia, it was reported that TNF-α plays a crucial role in mediating lung injury [6], whereas corylin ameliorated LPS-induced pulmonary damage. The latter effect was due to the modulation of interleukin 6/signal transducer and activator of transcription 3; and mitogen-activated protein kinase pathways [7].

Orfanos’ group revealed that in patients with COVID-19-related acute respiratory distress syndrome (ARDS), soluble angiotensin converting enzyme 2 is upregulated, while endothelial nitric oxide synthase expression is reduced [8]. The mechanisms by which histone deacetylases’ inhibition abrogates pleural fibrosis were also investigated [9] to demonstrate that the SA-5-Dox-LP liposome increases doxorubicin’s therapeutic efficiency in lung cancers overexpressing HER2 [10]. It was also suggested that sPLA2-IIA is associated with the early diagnosis of acute respiratory distress syndrome (ARDS), contributing to our knowledge regarding the pathophysiology of the disease [11]. Furthermore, therapeutic targets in lung neuroendocrine neoplasms were also identified [12].

Endothelial barrier function examination revealed the protective role of unfolded protein response (UPR) activation due to tunicamycin in LPS-induced injury [13]. The molecular mechanisms mediating the protective effects of heat shock protein 90 (Hsp90) inhibition towards lung barrier dysfunction were also investigated [14]. Hsp90 inhibitors exert anti-inflammatory activities and induce UPR [15,16]

In addition to original research articles, three complementary review manuscripts were also published in our Special Issue. The authors summarized the most up-to-date information on COVID-19-related lung fibrosis [17], and COPD pathophysiology [18]. Moreover, further relevant information on the anti-fibrotic and anti-inflammatory actions of α-melanocytic hormone was also provided [19].

The articles included in our collection have been largely cited—and viewed—by our target audience. It is our great hope that the corresponding material will propel and inspire novel investigative and therapeutic avenues in pulmonology and lung pathophysiology. However, many important questions remain open, including those emerging from the topic of UPR-mediated endothelial barrier function, since hyperpermeability may lead to ARDS and death. This is important because UPR has been shown to be involved in barrier-protective activities and interrelates with P53. This tumor suppressor protein mediates the effects of Hsp90 inhibitors in the microvasculature, and it is affected by UPR fluctuations. NEK kinases—which regulate P53 phosphorylation—are induced in septic mice [20].

Furthermore, assessing the involvement of inositol-requiring enzyme-1α, protein kinase RNA-like ER kinase, and activating transcription factor 6 in the protective effects of growth-hormone-releasing hormone antagonists (GHRHAnt) in lung disease should provide exciting information regarding the anti-inflammatory and anti-oxidative activities of these peptides in a diverse variety of disorders. It is known that UPR is involved in those effects, but more information regarding this particular context is needed [20].

To conclude this editorial synopsis, we would like to emphasize the urgent need to uncover the exact signaling pathways that regulate lung barrier function. New findings and information will assist in the development of targeted medical countermeasures to treat lung disease and will contribute to preventing infectious respiratory disorders. To paraphrase Socrates’ reflections in *Phaedrus*—written by Plato (≈370 BC)—knowledge delivers the actual wealth form, namely wisdom.
